# Intestinal obstruction due to small intestinal metastasis from primary Merkel cell carcinoma of the gluteal region

**DOI:** 10.3332/ecancer.2022.1493

**Published:** 2022-12-19

**Authors:** Juan Francisco Olivos-Gonzales, Elily Apumayta-Requena, Andrés Guevara-Jabiles, Mercedes Bravo-Taxa

**Affiliations:** 1 Fellow in Abdominal Surgical Oncology, Instituto Nacional de Enfermedades Neoplásicas, Lima 15038, Peru; 2 Surgical Oncology Resident, Instituto Nacional de Enfermedades Neoplásicas, Lima 15038, Peru; 3 Department of Abdominal Surgery, Instituto Nacional de Enfermedades Neoplásicas, Lima 15038, Peru; 4 Department of Oncologic Pathology, Instituto Nacional de Enfermedades Neoplásicas, Lima 15038, Peru; a https://orcid.org/0000-0002-0559-0295; b https://orcid.org/0000-0002-1828-7009; c https://orcid.org/0000-0001-9427-0068; d https://orcid.org/0000-0002-6965-4841

**Keywords:** carcinoma, Merkel cells, intestinal obstruction, neoplasm, neoplastic metastasis, neoplasm recurrence

## Abstract

Merkel cell carcinoma (MCC) is a rare neoplasm of unknown multifactorial origin first described in 1972. It occurs most often in older Caucasian males and is typically associated with sun-exposed areas of skin. However, cases have also been reported in other areas, such as the trunk and the gluteal region. Metastatic disease will occur in up to one-third of cases at onset or during the course of the disease, including metastases to the abdominal organs. We present the case of a 53-year-old male with a history of primary MCC of the right buttock and local resection surgery. Eighteen months later, he presented with a small bowel obstruction and had an emergency segmental bowel resection. Pathology examination with immunohistochemistry concluded that findings were consistent with metastatic MCC.

## Introduction

Merkel cell carcinoma (MCC) is a rare malignant neuroendocrine neoplasm first described by Toker [1] in 1972. MCC occurs mainly in sun-exposed areas due to its association with ultraviolet radiation, but it is also associated with immunosuppression and Merkel cell polyomavirus infection.

Merkel cell polyomavirus infection is detected in some healthy adults. Serological testing indicates that infection develops in childhood and is asymptomatic in nearly all cases, but some infected people develop polyomavirus-associated MCC. This association is found in 80% of cases, with polyomavirus genomic sequences integrated into MCC tumour DNA [18].

There are no reports of precursor, premalignant lesions and MCC presents as localised disease in more than half of cases. Fewer than 10% of patients have metastatic MCC at diagnosis [2]. Metastases occur mainly in the abdominal organs, lymph nodes, lungs, bones and brain [3, 4]. In 2010, metastasis to the abdominal organs was reported in up to 17 cases, most commonly in the stomach [3]. Intestinal metastasis is rare, and its main manifestation is obstruction. We present the case of a patient with a history of MCC of the right buttock, who came to the Emergency Oncology Department and was treated for acute intestinal obstruction.

**Clinical case**

A 53-year-old male with a history of MCC of the right buttock classified as Stage IIA (pT3cN0M0) under the Eighth Edition of the American Joint Committee on Cancer [17]. This patient had surgery at another hospital 18 months before coming to our main hospital. Pathologic features were an 8.5 cm tumour, lymphovascular infiltration and a deep margin 0.5 mm from the tumour, with the remaining margins negative.

**Figure 1.**
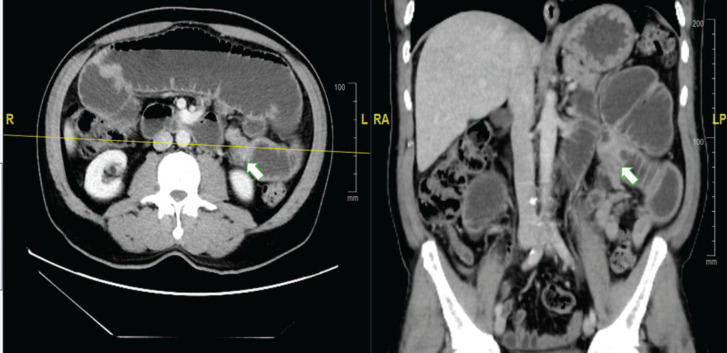
Abdominal CT scan with contrast. (a): Axial section. (b): Coronal section. The arrow indicates the point of tumorous intestinal stenosis, causing retrograde dilation of the proximal small intestinal loops.

The patient came to the Emergency Oncology Department after 1 day of epigastric cramps that had worsened over the previous 12 hours, associated with nausea, intermittent vomiting and intolerance of solids and liquids. Upon physical examination, a 5 cm mass was palpated in the mesogastrium, with bowel movements. The scar in the right gluteal region was in good condition, with no lesions. An abdominopelvic CT scan showed a hypodense, heterogeneous tumour in the mesentery, with irregular edges and contrast uptake, and measuring 32 × 45 × 53 mm. There was apparent jejunum infiltration, causing dilation of the proximal small intestinal loops and intestinal obstruction (Figure 1).

**Figure 2.**
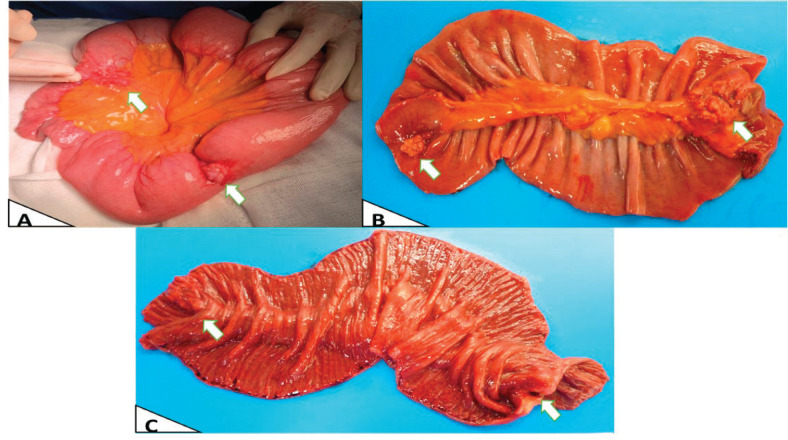
Macroscopy. (a): Intraoperative in vivo finding, showing the largest area with obstruction due to stenosis. (b): External surface of the surgical specimen opened and laid flat post-resection. (c): Intraluminal surface of the surgical specimen opened and laid flat, showing involvement of the entire intestinal wall and intestinal mucosa.

The patient had emergency surgery, which found two multi-lobed fibrous lesions attached to the small intestinal mesentery of the jejunum. One lesion measured 4 × 2 × 2 cm and was 1.5 metres from the angle of Treitz. It infiltrated and caused concentric stenosis of the jejunal loop. The second lesion measured 2 × 2 cm and was 50 cm cephalic to the first and was partially attached to the intestinal lumen (Figure 2). Both lesions caused substantial dilation of the proximal small intestinal loops.

**Figure 3.**
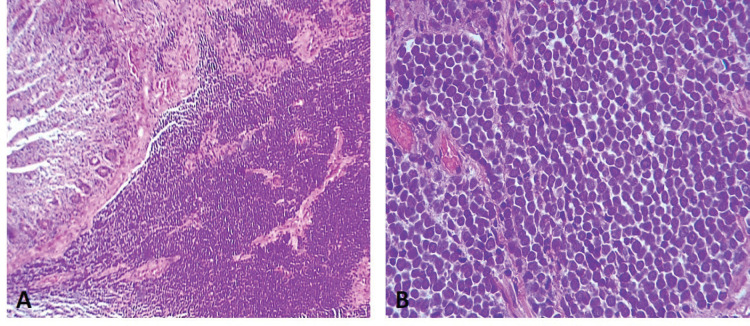
(a): Diffusely infiltrative tumour within the jejunal mucosa (H-E 100×). (b): Tumour cells are small, blue and uniformly round in shape. They have a high nuclear-to-cytoplasmic ratio, round-to-oval hyperchromatic nuclei with finely dispersed chromatin (salt and pepper), indistinct nucleoli, mitosis and conspicuous apoptotic bodies (H-E 400×).

It was decided to resect both tumours by performing segmental resection of the small intestine and mesentery, with primary isoperistaltic latero-lateral anastomosis. Postoperative progression was favourable and the patient was discharged 5 days after surgery, with no complications. The final pathology report concluded that both lesions were consistent with metastatic MCC (Figure 3), with involvement of the serous membrane of the jejunal wall but no lymphovascular or perineural invasion. None of the four resections involved lymph nodes and resection margins were clear. The patient is currently in follow-up and has no digestive discomfort.

## Discussion

In theory, MCC originates in Merkel cells in the dermal-epidermal junction, which form in the epidermis, have neuroendocrine characteristics and play a neurosensory role [5]. MCC incidence varies by race. There are 0.23 cases per 100,000 people in Caucasian populations, which is 20 times greater than in Afro-Caribbean populations [6]. MCC mainly affects males, with an average age of 69 years at diagnosis. Reported global 1-, 2- and 3-year survival rates are 88%, 72% and 55%, respectively [7]. MCC commonly presents on sun-exposed areas of skin, with firm, painful nodules that grow gradually. However, MCC has also been described in areas that are not sun-exposed, such as the trunk and gluteal region [8].

Polyomavirus-associated MCC in areas that are not sun-exposed is more common in younger patients (as in our case) and in females. Polyomavirus-associated tumours have viral DNA integrated into the tumour genome, preserving the expression of viral proteins that can strengthen the virus. There are also reports that patients with polyomavirus-associated MCC have a better prognosis than polyomavirus-negative patients [18].

**Figure 4.**
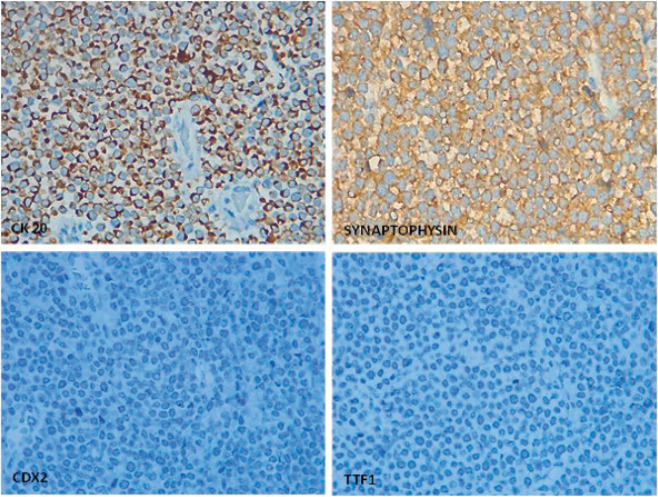
Classic perinuclear dot-like pattern for CK20 expression and cytoplasmic synaptophysin positivity in MCC whilst TTF1 and CDX2 are negative for tumour cells.

Histological diagnosis of MCC requires immunohistochemical tumour markers, which have a neuroendocrine nature. Neuron-specific enolase, chromogranin A, synaptophysin and proconvertases PC1/PC3 and PC2 have been identified as tumour markers that differentiate MCC from non-neuroendocrine neoplasms like malignant melanoma, lymphomas, leukaemias and small-cell carcinomas [14]. MCC is negative for markers such as S100, HMB-45 and specific cytokeratins. Moreover, the CD44 marker has been described as a risk factor for metastatic behaviour [5, 15]. Primary tumour immunohistochemistry for our patient was positive for CK20, epithelial membrane antigen and synaptophysin. However, the intestinal metastasis was positive for CK20 and negative for TTF-1 and CDX-2. The last two immunohistochemical markers make it possible to differentiate small-cell carcinomas that can be positive for CK20 and negative for TTF-1 (Figure 4) [5, 7, 15].

To complete staging, performing a PET scan with 90% sensitivity and 98% specificity to identify distant metastasis or lymph node macrometastasis is recommended. This step can change therapeutic measures in up to 27% of cases [2]. National Comprehensive Cancer Network Guidelines also suggest performing CT scans of the neck, thorax, abdomen and pelvis if PET scans find regional lymph node metastasis [9]. A sentinel lymph node biopsy (SLNB) is also recommended to complete staging in patients with clinically lymph node-negative MCC because one third of patients will have microscopic involvement. The specific 5-year survival rate varies from 86% with a negative SLNB to 64% with a positive SLNB [2, 9]. Our patient did not have an SLNB or a PET scan for initial staging.

Metastatic MCC accounts for up to one-third of all cases at onset and over the course of the disease, with an average of 24 months to recurrences [2]. Our case presented metastasis earlier. Metastasis is most common in the abdominal organs (51%) and lymph nodes (27%), and less common in the lungs (10%), bones (10%) and brain (3%–6%) [4, 6, 10]. Evidence of recurrent and/or metastatic disease is scarce. Song *et al* [4] reported that metastasis will occur in 40% of patients within 1 year of a Stage I, II or III MCC diagnosis, in 73% of patients within 3 years and in 100% of patients within 5 years. The first case of intra-abdominal involvement, metastasis to the colon, was reported in 1984, with cases of metastasis to the small intestine reported later [11, 12]. A case of generalised metastasis to the upper gastrointestinal tract, found at autopsy, has also been reported [13]. In our case, secondary intestinal lesions manifested clinically as a profile of intestinal obstruction after 12 months of treatment for the main MCC lesion.

There was no global consensus on treating and monitoring MCC until 2009, when the NCCN published recommendations for managing the disease, which vary by clinical stage [2, 9]. Under these recommendations, patients with localised disease have wide local excision surgery (with margins of 1–2 cm) and an SLNB. Adjuvant radiotherapy (RT) is not recommended if the SLNB is negative, the tumour is smaller than 1 cm and the patient has no risk factors (positive or inadequate margins, lymphovascular invasion, head or neck tumours, immunocompromise) due to the low, 6% risk of local recurrence in this scenario [2]. Patients who do not meet these guidelines should receive adjuvant RT at the primary site after surgery. Patients with a positive SLNB or metastatic MCC should be referred to a specialist centre to discuss next steps for surgical or RT treatment combined with systemic therapy [2, 9].

MCC is a rare disease and there is little evidence from prospective studies about non-surgical treatment of locally advanced or metastatic disease. However, new, early-phase clinical trials are testing the use of systemic agents combined with immunotherapy as first- and second-line treatment of MCC that is locally advanced and not treatable by initial surgical resection and metastatic MCC [16]. The use of three agents – two PD-1 inhibitors (pembrolizumab and nivolumab) and a PD-L1 inhibitor (avelumab) – has been approved by the U.S Food and Drug Administration.

Our patient had multiple risk factors and did not have an SLNB. After 18 months, systemic recurrence in the intra-abdominal organs was confirmed, showing the importance of adequate staging at diagnosis.

## Conclusion

MCC is a rare cutaneous neoplasm that can metastasise to the abdominal organs. A diagnosis or history of MCC means the disease should be considered a possible cause of intestinal obstruction.

## Conflicts of interest

The authors declare there are no conflicts of interest with the publication of this case report.

## Source of funding

This research has not received any funding.
